# Comparative performance analysis of K-nearest neighbour (KNN) algorithm and its different variants for disease prediction

**DOI:** 10.1038/s41598-022-10358-x

**Published:** 2022-04-15

**Authors:** Shahadat Uddin, Ibtisham Haque, Haohui Lu, Mohammad Ali Moni, Ergun Gide

**Affiliations:** 1 School of Project Management, Faculty of Engineering, The University of Sydney, Forest Lodge, NSW 2037 Australia; 2 School of Electrical and Information Engineering, Faculty of Engineering, The University of Sydney, Darlington, NSW 2008 Australia; 3 School of Health and Rehabilitation Sciences, Faculty of Health and Behavioural Sciences, The University of Queensland, St Lucia, QLD 4072 Australia; 4 School of Engineering and Technology, CQUniversity (Sydney), Sydney, NSW 2000 Australia

**Keywords:** Diseases, Applied mathematics

## Abstract

Disease risk prediction is a rising challenge in the medical domain. Researchers have widely used machine learning algorithms to solve this challenge. The *k*-nearest neighbour (KNN) algorithm is the most frequently used among the wide range of machine learning algorithms. This paper presents a study on different KNN variants (Classic one, Adaptive, Locally adaptive, k-means clustering, Fuzzy, Mutual, Ensemble, Hassanat and Generalised mean distance) and their performance comparison for disease prediction. This study analysed these variants in-depth through implementations and experimentations using eight machine learning benchmark datasets obtained from Kaggle, UCI Machine learning repository and OpenML. The datasets were related to different disease contexts. We considered the performance measures of accuracy, precision and recall for comparative analysis. The average accuracy values of these variants ranged from 64.22% to 83.62%. The Hassanaat KNN showed the highest average accuracy (83.62%), followed by the ensemble approach KNN (82.34%). A relative performance index is also proposed based on each performance measure to assess each variant and compare the results. This study identified Hassanat KNN as the best performing variant based on the accuracy-based version of this index, followed by the ensemble approach KNN. This study also provided a relative comparison among KNN variants based on precision and recall measures. Finally, this paper summarises which KNN variant is the most promising candidate to follow under the consideration of three performance measures (accuracy, precision and recall) for disease prediction. Healthcare researchers and stakeholders could use the findings of this study to select the appropriate KNN variant for predictive disease risk analytics.

## Introduction

The *k*-nearest neighbour (KNN) algorithm is a supervised machine learning algorithm predominantly used for classification purposes. It has been used widely for disease prediction^1^. The KNN, a supervised algorithm, predicts the classification of unlabeled data by taking into account the features and labels of the training data^2^. Generally, the KNN algorithm is able to classify datasets using a training model similar to the testing query by taking into account the *k* nearest training data points (neighbours), which are the closest to the query it is testing. Finally, the algorithm performs a majority voting rule to check which classification to finalise. Among all machine learning algorithms, the KNN algorithm is one of the simplest forms and is widely used in classification tasks because it has a very adaptive and easy-to-understand design^3^. The algorithm is renowned for its usage in solving regression and classification challenges for data of different sizes, label numbers, noise levels, ranges, and contexts^4^. Thus, this paper is forming a study around this algorithm based on classifying medical datasets, as predicting diseases is a real-world challenge. It is compelling to identify how it can adapt to aid this problem.

The algorithm is simplistic in its workings and calculations. It gives itself options to be modified in various aspects to decrease its limitations and challenges and increase its accuracy and applicability to be used in a wider variety of datasets. The classic KNN algorithm suffers from various limitations that abate its classification prowess, such as being unbiased to all its classification-dependent neighbours, lack of distance calculation features between data points, and taking into account unnecessary dataset features^5^. However, as KNN is adaptable to numerous modifications, it gives rise to different KNN forms or variants. The KNN variants differ in various algorithmic aspects, such as optimising the *k* parameter, improving distance calculations, adding weight to different data points, and truncating training datasets to resolve the challenges mentioned earlier^6^.

From the wide variety of research papers proposing different variants, the lion’s share of the KNN variants focuses on creating optimal *k* values. Wettchereck et al.^7^ and Sun and Huang^8^ proposed an algorithm called the adaptive KNN. They proposed training the training dataset itself to find the *k* value for each training dataset within a limited range. Then, through the *k* values of the training data, the testing data are classified to obtain the closest training data and attain its *k* value, through which it restarts its classification based upon the obtained parameter. While they proposed a widely adaptive *k* value finding a formula, Pan et al.^9^ proposed a super variation of the adaptive KNN called the locally adaptive KNN based on the discrimination class. This paper proposes a locally adaptive approach that considers multiple ranking methodologies. The paper states how their proposed algorithm decreases the limitation of only taking into account the majority classes by considering the minority classes and calculating an optimal *k* value through multiple probabilistic ranking formulae. There are also *non*-parametric KNN variants, such as the algorithm proposed by Cherif et al.^10^, that focus on finding optimal *k* values. They proposed a combination of two different algorithms to reduce the need to find an optimal *k* value. Their algorithm uses the *k*-means algorithm, which truncates the dataset into cluster points, and then runs the classic KNN algorithm to find the one nearest neighbour for the final classification. Another KNN variant that focuses on combining multiple algorithms to remove the need to find the optimal *k* value is the variant proposed by Hassanat et al.^11^, where the authors detail out a KNN algorithm with an ensemble approach. Their algorithm removes the need for a *k* parameter, as it performs iterative classifications using *k* values of a limited range.

Other than the variants focusing on finding the optimal *k* values, others focus on different internal aspects to improve accuracy. The KNN variant introduced by Han et al.^12^ and Yigit^13^ is an algorithm that focuses on weight attribution. Their algorithms take into account the weight factors of each nearest neighbour according to their distance and class frequency. These accounts decrease the limiting factor of taking all *k* nearest neighbours equally and increase the chances of the algorithm in predicting final classifications. Another KNN variant that focuses on weight attribution is the weighted mutual KNN algorithm proposed by Dhar et al.^14^. The algorithm works by truncating the training dataset into only mutual sets and running the testing dataset through it to classify the output from the nearest mutual neighbours. Their algorithm helps remove the noise from the training dataset and add weight attributes for the final classification. Keller et al.^15^, on the other hand, introduce an extra mathematical addition to the classic KNN algorithm, known as fuzzy sets. Their proposed algorithm, fuzzy KNN, focuses on membership assignment. The membership assignments are a different form of weight attribution, which calculates the probabilistic chances of a neighbour class becoming the final classification. Alkasassbeh et al.^16^ detailed another type of KNN variant that centres the point of attention on regular distance metrics. The paper states the usages of a new distance metric called Hassanat distance, which proves to be more efficient in classifying datasets than the traditional metrics of Euclidean and Manhattan distances. Another distance focusing KNN variant is the variant proposed by Gou et al.^17^. Their variation is different from the previous paper, as they do not propose a new distance metric but an algorithm that enhances the outputs of any distance formulae being used. The paper proposes generalised mean distance calculations and vector creations for the nearest neighbours of each different class. The algorithm is said to remove the limitations of being unbiased to weight attributions and enhance the accuracies by using their local mean vector calculations.

As discussed above, while most KNN variants focus on finding the optimal *k* values, other variants emphasise the overall classification accuracy. Each variant has its unique design and rationale. Each revealed the best performance in the corresponding study that first introduced it to the literature. No previous research attempts to make a performance comparison among different KNN variants. Therefore, to fill this gap, it is required to make a comparative performance analysis of these variants using the same datasets and experimental setup. By considering the parameter values leading to the best performance for each variant, this study used eight different disease datasets to compare the performance of 10 KNN variants.

## K-nearest neighbour algorithm and its different variants

### The Classic KNN Algorithm

The classic KNN algorithm is a supervised machine learning algorithm that is predominantly used for classification purposes^18^. The algorithm consists of a variable parameter, known as *k*, which translates to the number of ‘nearest neighbours’. The KNN algorithm functions by finding the nearest data point(s) or neighbour(s) from a training dataset for a query. The nearest data points are found according to the closest distances from the query point. After locating the *k* nearest data points, it performs a majority voting rule to find which class appeared the most. The class that appeared the most is ruled to be the final classification for the query.

**Figure 1:**
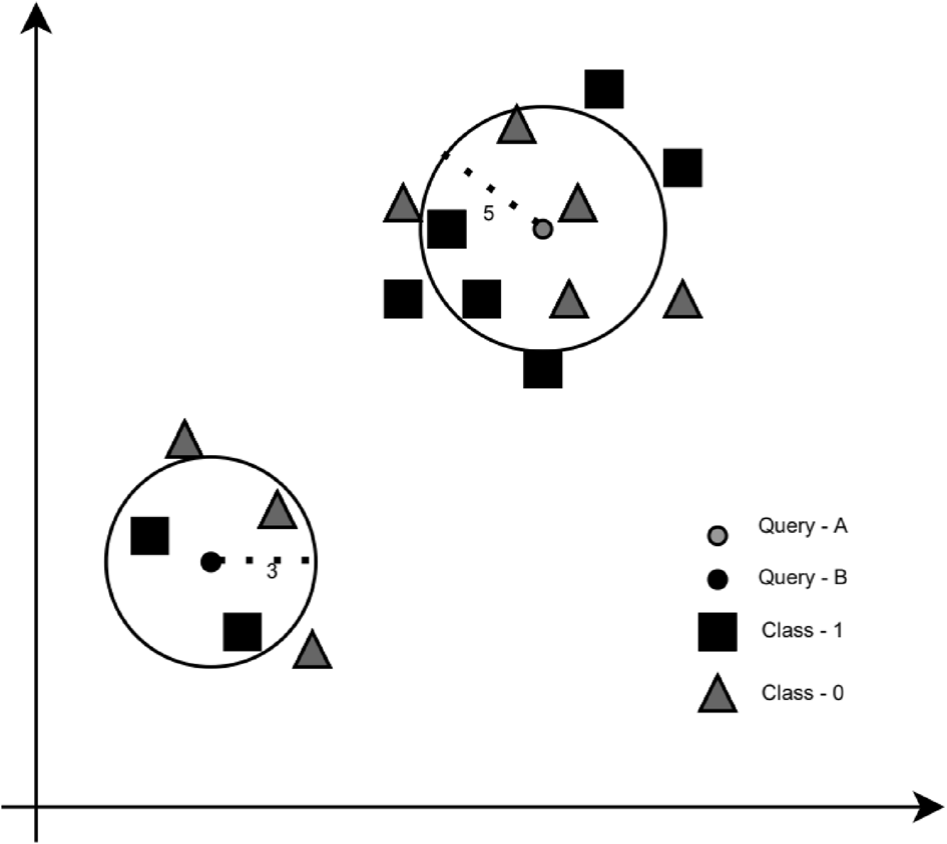
Visual illustration of the KNN algorithm.

Figure 1 illustrates an example. As *k* is 3 for Query B, it searches for the 3 nearest neighbours and finds that from the 3 nearest neighbours, two are of class 1, and 1 is of class 0. It then uses the majority voting rule to classify its class as 1. Similarly, as *k* is 5 for Query A, as there are a greater number of neighbours that are characterised as Class 0, it classifies its class as 0.

### KNN variants considered in this study

#### Adaptive KNN (A-KNN)

The adaptive KNN algorithm is a variant that focuses on selecting the optimal *k* value for a testing data point^7,8^. It works by implementing a separate algorithm to determine the optimal *k* value for each data point of the training dataset. The main algorithm then finds the nearest neighbour from the training dataset and inherits its *k* value for a given testing data point. This KNN variant proceeds to function as the classic KNN algorithm to predict the output using this inherited *k* value.

#### Locally adaptive KNN with Discrimination class (LA-KNN)

This variant considers information from discrimination classes to determine the optimal *k* value. The discrimination class concept considers quantity and distribution from the majority class neighbours and the second majority class neighbours in the *k*-neighbourhood of a given testing data point^9^. The algorithm uses various steps to define discrimination classes. After selecting one of those classes, it proceeds to form a ranking table with different *k* values, distances from centroids and their ratio. From the table, it follows a ranking process to output the optimal *k* value.

#### Fuzzy KNN (F-KNN)

The fuzzy KNN algorithm revolves around the principle of membership assignment^15^. Similar to the classic KNN algorithm, the variant proceeds to find the *k* nearest neighbours of a testing dataset from the training dataset. It then proceeds to assign “membership” values to each class found in the list of *k* nearest neighbours. The membership values are calculated using a fuzzy math algorithm that focuses on the weight of each class. The class with the highest membership is then selected for the classification result.

#### K-means clustering-based KNN (KM-KNN)

The clustering-based KNN variant involves the combination of two popular algorithms: *k-*means and 1NN^10^. This variant uses the *k*-means algorithm to cluster the training dataset according to a preset variable (number of clusters). It then calculates the centroids of each cluster, thus making a new training dataset that contains the centroids of all the clusters. The 1NN algorithm is performed on this new training dataset, where the single nearest neighbour is taken for classification.

#### Weight adjusted KNN (W-KNN)

This version of the KNN algorithm focuses on applying attribute weighting. This algorithm first assigns a weight to each of the training data points by using a function known as the kernel function^12^. This weight assignment aims to give more weight to nearer points while giving less weight to faraway points. As the distance increases, any function that decreases the value can be used as a kernel function. The frequency of all nearest neighbours is then used to predict the output class of a given testing data point. This KNN variant considers the classification importance of different attributes in defining the kernel function for a multiattribute dataset.

#### Hassanat distance KNN (H-KNN)

The Hassanat KNN algorithm is a variant that has its focal point on the distance measurement formula. This variant follows the simple design of the KNN algorithm, but it proposes an advanced way to find the distance between two data points^16^. The new distance formula is called the Hassanat distance, and it revolves around the usage of maximum and minimum vector points, similar to weight attributions in other variants. The Hassanat distance metric of this variant calculates the nearest neighbours of a testing query and performs the majority voting rule, similar to the classic KNN algorithm.

#### Generalised mean distance KNN (GMD-KNN)

The generalised mean distance KNN or GMD-KNN is a variant that revolves around the principle uses of local vector creations and repeated generalised mean distance calculations 17. The algorithm works by first storing sorted lists of *k* nearest neighbours for each class. It then proceeds to convert each list to local mean vectors, from which multiple iterative mean distance calculations are computed to output a final value for the distance of each class to the testing query. The class with the smallest distance is then deemed to be the correct prediction for the testing query.

#### Mutual KNN (M-KNN)

The mutual KNN algorithm focuses on the principle of mutual neighbours^14^. The algorithm first transforms the training dataset by removing sets that have no mutual *k* nearest neighbours with the other sets. This creates a truncated training dataset that consists of less noise and anomalies. The algorithm then uses the testing dataset to find the *k* nearest neighbours from the training dataset and finds the *k* nearest neighbours of the testing datasets nearest neighbours. This allows the algorithm to determine the mutual nearest neighbours, which can be assessed as a candidate for classification. The testing datasets are classified using the majority voting rule.

#### Ensemble approach KNN (EA-KNN)

The KNN variant EA-KNN is based on an ensemble approach to remove the problem of having a fixed “*k*” parameter for classification. This algorithm works by using a Kmax value of $\sqrt n$, with n being the size of the training dataset, to find the k-nearest neighbours of a testing query^11^. It then sorts the list of nearest neighbours according to the distance and performs weight summation operations on it. The weight summation operations are performed by iteratively adding an inverse logarithm function for “*k*” values starting from 1 to Kmax in increments of 2. The class with the largest weight summation is then deemed to be the predicted classification for the testing query.

## Methods

### Research datasets

The research that is being undertaken is based upon one primary domain, medical domains, and other secondary domains, which are purely random, to eliminate bias. Table 1 presents the datasets that are being used in this study and their respective attributes in terms of the number of features, data size and domain. They were taken from Kaggle^19^, UCI Machine Learning Repository^20^ and OpenML^21^. The datasets have different characteristics in terms of features, attributes, and sizes, and most belong to the medical domain for the relevance of disease risk prediction.

**Table 1:** A brief list of eight disease datasets considered in this study.

ID	Datasets	Features	Data size	References
D1	Heart Attack Possibilities	13	303	Bhat^22^
D2	Heart Failure Outcomes	12	299	Chicco et al.^23^
D3	Diabetes	8	768	Mahgoub^24^
D4	Heart Disease Prediction	13	270	Bhat^22^
D5	Chronic Kidney Disease Preprocessed	24	400	Soundarapandian^25^
D6	Chronic Kidney Disease Prediction	13	400	Soundarapandian^25^
D7	Pima Indians Diabetes	8	767	Smith et al.^26^
D8	Breast Cancer	5	569	Suwal^27^

### Performance comparison measures

#### Confusion matrix

**Figure 2:**
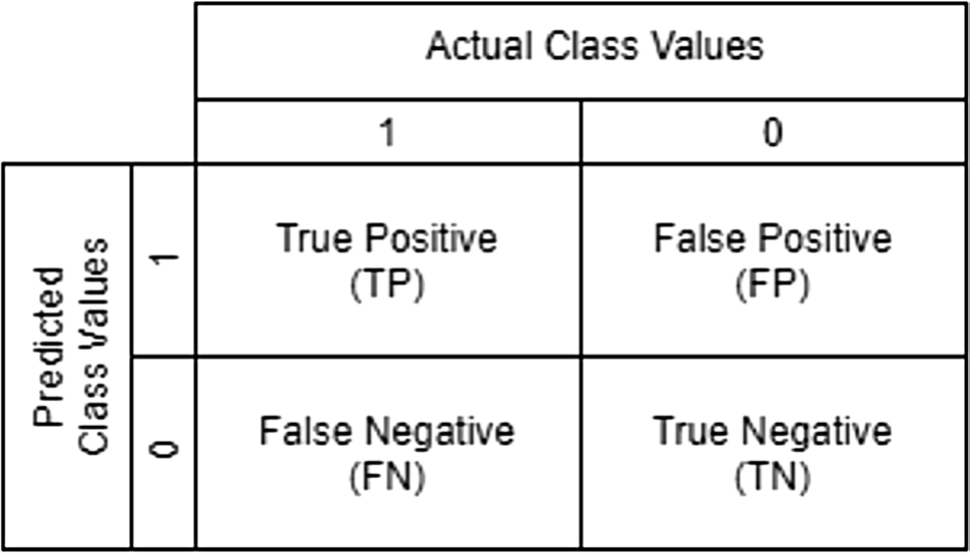
Confusion Matrix.

The performance measures used to analyse the results are academically renowned and revolve around the usage of the confusion matrix^28^. Figure 2 presents the visual for the matrix. The matrix is the amalgamation of results from classifications and has four primary attributes that present the result data. If the classification is predicted to be 1 and the true value is 1, the result is classified as true positive (TP). The same principle revolves around the value 0 and is classified as true negative (TN). When the prediction is 1 and the true value is 0, the result is classified as false positive (FP), with the inverse being called false negative (FN).

In this research, the confusion matrix is used to create three performance measures: accuracy, precision and recall.

The accuracy measure is calculated by taking all the true predictions and dividing them among all the predicted values, including the true predictions.

$$Accuracy = \frac{TP + TN}{{TP + TN + FP + FN}}$$

where TP, TN, FP and FN are the true positive, true negative, false positive and false negative cases of the result data, respectively.

The precision measure is calculated by taking the true positive values and dividing them among both true and false positive values.

$$Precision = \frac{TP}{{TP + FP}}$$

where TP and FP are the true positive and false positive cases of the result data, respectively.

The recall measure is calculated similarly to the precision measure by taking the true positive values and dividing them among the true positive and false negative values.

$$Recall = \frac{TP}{{TP + FN}}$$

where TP and FN are the true positive and false negative cases of the result data, respectively.

These three performance measures will be used to assess the classification results of the variants implemented in this paper. The complete set of these three performance measures will be used to create a new measure that will be discussed in the next section.

#### Relative performance index (RPI)

The relative performance index is a breakthrough assessor that collects data results of any other measure (accuracy, precision and recall, etc.) and produces a probabilistic result for the final assessment. The new performance measure that is being proposed here is inspired by another RPI measure proposed by Nagle^29^. The author proposed a new probabilistic calculation that removes bias by considering the range of results produced by a particular field and extracting the number of times the results of that field were above other fields. The RPI for a field is calculated using the extracted values and the number of fields that exist.

The following equation describes the new performance measure of this study:

$$Relative \;Performance \;Index \left( {RPI} \right) = \sum\limits_{i = 1}^{d} {\frac{{\left( {a_{i} - a_{i}^{*} } \right)}}{d}}$$

where $a_{i}^{*}$ is the minimum accuracy/precision/recall value among all variants for dataset $i$, $a_{i}$ is the accuracy/precision/recall value for the variant under consideration for dataset $i$, and $d$ is the number of datasets considered in this study.

A higher RPI value indicates prediction superiority considering the underlying performance measures (e.g., accuracy and precision) and vice versa.

## Results

**Figure 3:**
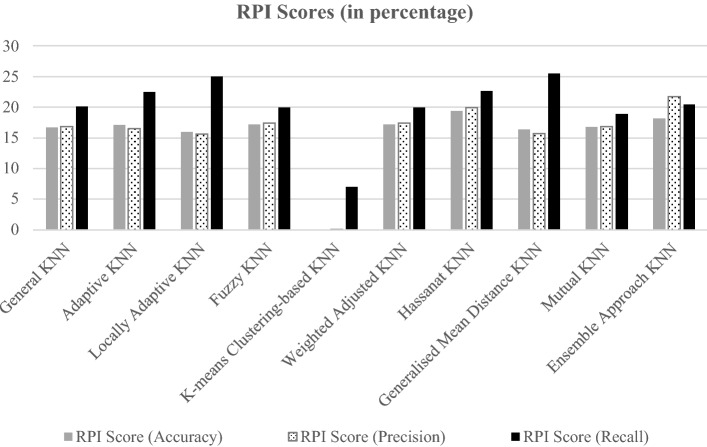
Average relative performance index (RPI) scores for the three performance measures.

The variants that require a *k* parameter were tested using *k* values of 1, 3, 5, 7 and 9. For this results section, the results of the *k* values with the highest performance in each variant were selected. The accuracy, precision and recall of the different KNN variants based on the research datasets are presented in Tables 2, 3 and 4, respectively. Table 5 shows the number of times a variant results in having the highest measure, and Fig. 3 introduces the RPI scores of each measure in average values.

**Table 2:** Accuracy (%) comparison among KNN variants.

Dataset ID	Classic KNN	Adaptive KNN	Locally adaptive KNN	Fuzzy KNN	K-means clustering-based KNN	Weight adjusted KNN	Hassanat KNN	Generalised mean distance KNN	Mutual KNN	Ensemble approach KNN
D1	76.35	73.64	69.59	73.65	39.86	73.65	**85.14**	69.59	71.62	77.03
D2	58.87	63.83	58.87	63.83	47.52	63.83	67.38	62.41	65.96	**68.79**
D3	75.25	75.00	75.51	74.24	68.18	74.24	76.26	74.24	74.24	**79.29**
D4	79.51	78.69	76.23	**81.97**	68.85	**81.97**	80.33	77.05	80.33	81.15
D5	96.26	96.26	**98.40**	95.72	67.38	95.72	96.79	**98.40**	95.72	93.05
D6	96.92	97.44	**97.95**	97.44	64.10	97.44	96.41	97.44	94.87	92.31
D7	73.88	73.60	75.00	73.60	68.82	73.60	75.56	74.44	74.44	**76.69**
D8	90.38	**92.10**	90.03	91.07	89	91.07	91.07	91.41	90.72	90.38
Average	80.93	81.32	80.20	81.44	64.22	81.44	**83.62**	80.62	80.99	82.34

**Table 3:** Precision (%) comparison among different KNN variants.

Dataset ID	Classic KNN	Adaptive KNN	Locally adaptive KNN	Fuzzy KNN	K-means clustering-based KNN	Weight adjusted KNN	Hassanat KNN	Generalised mean distance KNN	Mutual KNN	Ensemble approach KNN
D1	80.90	77.42	77.11	76.84	51.52	76.84	**86.96**	77.11	73.53	78.57
D2	39.13	46.51	42.65	46.15	34.15	46.15	52.78	46.27	50	**54**
D3	62.16	60.32	60.61	61.17	49.12	61.17	63.48	58.91	60.75	**74.71**
D4	82.98	81.25	77.55	**85.42**	75	**85.42**	84.78	78	78.57	85.11
D5	**100**	**100**	**100**	**100**	67.38	**100**	**100**	**100**	**100**	**100**
D6	**100**	99.18	98.40	**100**	64.10	**100**	99.17	98.39	**100**	**100**
D7	60	58.49	60	59.78	50.75	59.78	62.63	58.97	62.07	**71.64**
D8	98.98	98.06	97.52	**98.99**	97.49	**98.99**	**98.99**	97.12	**98.99**	98.98
Average	78.02	77.65	76.73	78.54	61.19	78.54	81.10	76.85	77.99	**82.88**

**Table 4:** Recall (%) comparison among different KNN variants.

Dataset ID	Classic KNN	Adaptive KNN	Locally adaptive KNN	Fuzzy KNN	K-means clustering-based KNN	Weight adjusted KNN	Hassanat KNN	Generalised mean distance KNN	Mutual KNN	Ensemble approach KNN
D1	80	80	71.11	81.11	18.89	81.11	**88.89**	71.11	83.33	85.56
D2	37.50	41.67	60.42	37.50	58.33	37.50	39.58	**64.58**	25	56.25
D3	55.20	60.80	**64**	50.40	22.40	50.40	58.40	60.80	52	52
D4	69.64	69.64	67.86	73.21	48.21	73.21	69.64	69.64	**78.57**	71.43
D5	94.44	94.44	97.62	93.65	**100**	93.65	95.24	97.62	93.65	89.68
D6	95.20	96.80	98.40	96	**100**	96	95.20	97.60	92	88
D7	50.89	55.36	**61.61**	49.11	30.36	49.11	55.36	**61.61**	48.21	42.86
D8	88.24	**91.40**	89.14	89.14	87.78	89.14	89.14	**91.40**	88.69	88.24
**Average**	71.39	73.76	76.27	71.27	58.25	71.27	73.93	**76.80**	70.18	71.75

**Table 5:** Comparison of KNN variants showing the number of times they presented the highest measurement values.

KNN Variants	Accuracy measure (#)	Precision measure (#)	Recall measure (#)
Classic KNN	0	2	0
Adaptive KNN	1	1	1
Locally Adaptive KNN	2	1	2
Fuzzy KNN	1	4	0
K-means Clustering-based KNN	0	0	2
Weight Adjusted KNN	1	4	0
Hassanat KNN	1	4	1
Generalised Mean Distance KNN	1	1	3
Mutual KNN	0	3	1
Ensemble Approach KNN	3	7	0

For the accuracy measure, according to Table 2, Hassanat KNN showed the highest average accuracy (83.62%), followed by the ensemble approach KNN (82.34%). However, according to Table 5, the ensemble approach KNN outputted the highest number of accuracies throughout the datasets (three times), followed by the locally adaptive KNN (two times). Regarding Fig. 3, Hassanat KNN had the highest accuracy score, followed by the ensemble approach KNN, with K-means clustering-based KNN showing the lowest score in terms of RPI accuracy.

Concerning the precision measure, according to Table 3, Ensemble Approach KNN showed the highest average precision (82.88%), followed by the Hassanat KNN (81.10%). The ensemble approach KNN outputted the highest precision in seven out of eight datasets, followed by Hassanat, weight adjusted and fuzzy KNNs (each with four times), according to Table 5. In Fig. 3, the ensemble approach KNN also obtained the highest RPI score regarding the precision measure, followed by the Hassanat KNN. The K-means clustering-based KNN variant also obtained the lowest RPI in this measure.

For the recall measure, as presented in Table 4, the generalised mean distance KNN showed the highest average recall (76.84%), followed by the locally adaptive KNN (76.27%). According to the results presented in Table 5, the highest number of times a KNN variant outputted the highest recall was the generalised mean distance KNN variant in three out of eight datasets, followed by locally adaptive and k-means clustering KNN (each with two times). As illustrated in Fig. 3, both the generalised mean distance and locally adaptive variants obtained the highest RPI score in the recall, followed by the locally adaptive variant. Finally, similar to previous RPI score measures, the K-means clustering-based KNN obtained the lowest score once more.

Table 6 presents the summary of each variant that resulted in the highest values in terms of its average, the number of times it showed the highest value and RPI scores for each of the three measures. This table provides essential insights into the potential candidate of the KNN variant to consider for disease prediction. For example, if the target performance measure of a research design is the average accuracy and RPI (accuracy), then the Hassanat KNN will be the most suitable one to consider. Similarly, one should consider the ensemble approach KNN if the target performance measure is precision.

**Table 6:** Summary of the different characterisations of measures of all KNN variants this study considered in terms of revealing the highest values.

KNN Variants	Accuracy			Precision			Recall		
	Average	# of times	RPI	Average	# of times	RPI	Average	# of times	RPI
**Classic KNN**									
Adaptive KNN									
Locally Adaptive KNN									
Fuzzy KNN									
Kmeans Clustering KNN									
Weight Adjusted KNN									
Hassanat KNN	**X**		**X**						
Generalised Mean Distance KNN							**X**	**X**	**X**
Mutual KNN									
Ensemble Approach KNN		**X**		**X**	**X**	**X**			

Although different variants perform differently across all datasets for the three performance measures considered in this study (from Tables 2, 3, 4), we did not find any statistically significant difference in their group-wise values when we applied the one-way ANOVA. Table 7 presents the results from the one-way ANOVA test. None of the significance values is ≤ 0.05.

**Table 7:** Results from the one-way ANOVA test for checking the significance of the difference of three performance measures across the ten KNN variants considered in this study.

		Sum of Squares	df	Mean Square	F	Sig
Accuracy	Between Groups	2200.496	9	244.500	1.594	0.134
	Within Groups	10,735.168	70	153.360		
	Total	12,935.664	79			
Precision	Between Groups	2468.842	9	274.316	0.670	0.733
	Within Groups	28,675.727	70	409.653		
	Total	31,144.569	79			
Recall	Between Groups	1915.288	9	212.810	0.424	0.918
	Within Groups	35,172.935	70	502.470		
	Total	37,088.223	79			

Finally, the advantages and limitations are distinguished and explained in Table 8.

**Table 8:** Comparison of KNN variants through advantages and limitations.

KNN Variant	Advantage(s)	Limitation(s)
**Classic KNN**	Low time complexity Can classify at high speeds compared to other machine learning algorithms	It does not consider minority class and weight of data points, which may cause the accuracy to fall for noisy datasets
Adaptive KNN (A-KNN)	Perform consistently better with small scale datasets	It does not provide the optimal *k* value, creating a disparity in choosing the optimal *k* values
Locally Adaptive KNN (LA-KNN)	It generally improves the classification performance by considering classes that are discriminated by the classic KNN properly rank the accuracies resulting from multiple *k* values	The variant is prone to a higher computational complexity than other variants The high time complexity makes the algorithm undesirable to be used for large scale datasets
Fuzzy KNN (F-KNN)	It considers class frequency and weight making it more probable in making a correct prediction	This variant does not provide an optimal *k* value, thus requiring additional changes in parameter settings
K-means Clustering-based KNN (KM-KNN)	The KM-KNN variant reduces the time complexity of the classic KNN algorithm by truncating the training dataset by forming clusters	The algorithm is unsuitable to noisy datasets as it clusters the training data Noisy datasets would produce uneven clusters and thus affect the classification process
Weight Adjusted KNN (WA-KNN)	It considers different *k* values for neighbourhood searching for a given query, making it more probable for greater accuracy	The algorithm discriminates the points which have a greater distance from the query, thus causing a bias
Hassanat KNN (H-KNN)	The H-KNN variant uses the Hassanat Distance metric, which allows the algorithm to measure the distance in terms of maximum and minimum vector points, making it prone to biased outcomes	It does not consider minority classes, which may affect its performance Inconsistent outcomes for noisy datasets
Generalised Mean Distance KNN (GMD-KNN)	It breaks the limitations of biasing the majority classes by considering all classes using a generalised distance algorithm formula It can eliminate biases resulting from variance in class weight and majority	It has many dependable variables, making it a high time complexity KNN variant
Mutual KNN (M-KNN)	The M-KNN variant removes noisy data from the dataset, thus improving the neighbourhood findings of the underlying query points and increasing the chances of correct classification	The algorithm incurs a high computational complexity cost due to its reiteration of nearest neighbour searches for training and the testing datasets This variant may be unsuitable for large scale datasets due to its high computational complexity
Ensemble Approach KNN (EA-KNN)	The Ensemble Approach variant involves using multiple *k* values within a suggestive range for the highest accuracy, thus removing the problem of inputting the optimal *k* parameter	If *k* takes a high number of values, the computational complexity will be an issue. Such complexity would make it undesirable to be used for large scale datasets

## Discussion

The experiments and the results from the performance measures were comprehensive in themselves. The tables provided in the results section show that most of the variants outperformed the classic or general KNN in terms of accuracy, precision and recall. The measures of the variants were taken as the *k* values of 1, 3, 5, 7 and 9. These values were used across all the KNN variants that required a *k* parameter to run. The *k* value that performed the best for each measure was selected for the comparison for each variant. Although it is best known to use $k = \surd n$, with n being the size of the dataset, for measuring performances^30^, the equal application of the same range of *k* values for each KNN variant returns the same non-biased results for a proper comparison. This study aims to compare the performance of each variant and not to output the optimal performance for each, as it would create biased results on the understanding that each algorithm runs optimally. Hence, the usage of equal parameter settings (i.e., considering the *k* value that generates optimal results) for each variant validates the juxtaposition of the performance measures.

The K-means clustering-based KNN variant performs the least in all aspects, even less than the classic KNN algorithm. This may be because the algorithm is formed around the principle of creating cluster centroids and using 1NN for the final classification. The research datasets are noisy and have outliers; thus, the low measures can be theorised to be due to the inaccurate creation of the clusters and their corresponding centroids. Additionally, the usage of 1NN only takes the nearest neighbour for classification, which is not enough to produce high accuracy. The algorithm could have been improved if a greater *k* value was used to increase the number of nearest neighbours to be used for the final majority voting rule. This would have decreased the bias from taking one nearest neighbour and enhanced the results in all three measures.

For choosing a variant to implement solely for accurate values, the Hassanat KNN variant is the most suitable option, as the improved distance metric of this variant proved its uniqueness and ability to handle data of different scales and noise levels very well. The ability to handle different scales of data proved well in this study, as the datasets that it was tested on were of all different types and scales of features. This variant might be ahead with respect to accuracy; however, it is lacking in terms of precision and recall results. The change in distance metrics is still not the optimal weight attribution replacement, and the variant is dependent on the *k* parameter. The variant can be theorised to be improved further if it consists of calculations to remove further noise from the training dataset or if it contains another step for better weight attribution during its majority voting rule process.

With regard to choosing a variant for improved precision values, the ensemble approach KNN variant is more fitting, as the variant is built on the principle of looking at class weights and consists of multiple iterations of different primary *k* values. The multiple iterations of primary *k* values abated one of the limitations of searching or needing an optimal *k* parameter. This variant’s unique design makes its classification highly precise. The variant is also not thus far off from producing high accuracy measures from the other variants, as seen from other result tables. The ensemble approach KNN algorithm could have been improved further if an optimal range of *k* values could have been found instead of constant primary values, as it would have increased its accuracy and precision measure results further.

Moreover, in terms of choosing a variant for greater recall, the generalised mean distance KNN variant would be the most appropriate selection. With its local vector and generalised mean distance calculations, this variant proves itself to work in generalising the need for weight attribution. Its unique characteristics make its classification results showcase the truest values on average throughout all the datasets. However, this variant is not high in other measures due to its variable *k* parameter. Not being able to find the optimal *k* parameter is a considerable limitation, which this variant does not consider. Knowing the optimal *k* parameter for each dataset can be theorised to increase the results of the other measures and improve the overall performance of this variant.

This study expectedly did not find any significant difference in performance measures for all variants (Table 7). All variants were intended to improve the baseline KNN when developed. An improvement of even 1% for any performance measures is considered a significant achievement for any classifications. However, such a slight difference would not make it statistically significant since the scale for each performance measure is 0–100%.

## Conclusion

Overall, Hassanat, ensemble approach and generalised mean distance can be selected as the most suitable KNN variants for disease prediction according to their high accuracy, precision and recall measures, respectively. These variants approached different limitations of the classic KNN and outperformed the rest in overall performance. Considering the top three performing variants, the individual measures of the three showcase that the ensemble approach KNN variant obtained average performance measurement values higher than the rest. This variant achieved the highest measurement precision and subsequently performed well in both accuracy and recall measurements. The ensemble approach KNN is the prime variant to be chosen among the rest. Its unique design for tackling multiple limitations proves to handle medical datasets most prominently in disease risk prediction. In general, most of the variants prove how effectively they can be used in the medical domain by presenting their abilities to obtain high-performance measures in a wide set of research datasets. The medical field consists of different scales and ranges of data. Overall, these variants demonstrated their capabilities to subside the general constraints and classify datasets in this domain. The variants are also adaptable, capable of further enhancement, and can be revamped to abate more general limitations.

Throughout the research analyses conducted in this study, the potential of one of the simplest machine learning algorithms can be viewed clearly. The KNN algorithm is an algorithm with many limitations. However, the research has shown how it also has one of the most adaptable designs. The variants considered in this study have presented results that prove how their algorithmic mutations can aid in solving problems by searching for optimal *k* values, adding better weight attributions, calculating local mean vectors of neighbours, truncating datasets to remove noise, taking into account mutual neighbours, and more. The study has shown how these variants can diminish the limitations and be used in various real-world classification purposes, especially in disease prediction. Disease risk prediction is a rising challenge, close to a grand challenge. The classification accuracies of this algorithm’s variants provide proof of their potential to be improved further to limit constraints.

In terms of future work, the variants concluded earlier can be studied further to be modified individually or merged. These variants can be integrated with one another, e.g., the generalised mean distance KNN can inherit the distance metric of the Hassanat KNN variant. This would allow the underlying variant to diminish more limitations than originally designed. These merged KNN variants can be studied based on disease risk classifications. Further variants, in addition to the variants considered in this study, can be studied as well in the context of disease risk prediction, with a greater number of medical datasets. The designs of KNN are versatile and are open to change. Medical domain-specific datasets are expanding; however, mutations of KNN have the capabilities to handle a wide variety of dataset characteristics. Last but not least, another possible future scope is to compare the best performing KNN variants with other related algorithms, such as reptile search algorithm^31^ and Aquila optimiser^32^, available in the literature for disease prediction.

## Data Availability

This study obtained research data from publicly available online repositories. We mentioned their sources using proper citations. The datasets analysed during the current study are available in the following repositories: Kaggle (https://www.kaggle.com/), UCI Machine learning repository (https://archive.ics.uci.edu/ml/index.php), OpenML (https://www.openml.org/).

## Abbreviations

KNN: K-nearest neighbour
A-KNN: Adaptive K-nearest neighbour
LA-KNN: Locally adaptive K-nearest neighbour
F-KNN: Fuzzy K-nearest neighbour
kM-KNN: K-means K-nearest neighbour
W-KNN: Weighted K-nearest neighbour
H-KNN: Hassanat K-nearest neighbours
GMD-KNN: Generalised mean distance K-nearest neighbour
M-KNN: Mutual K-nearest neighbour
EA-KNN: Ensemble approach K-nearest neighbour
TP: True positive
TN: True negative
FP: False positive
FN: False negative
RPI: Relative performance index
